# Healthcare-Associated Infections in a Cardiac Surgery Service in Brazil

**DOI:** 10.21470/1678-9741-2019-0284

**Published:** 2020

**Authors:** Guilherme Bail Ferreira, Juliana Carolina Sava Donadello, Leonardo Andrade Mulinari

**Affiliations:** 1 Universidade Federal do Paraná, Brazil.; 2 Department of Thoracic and Cardiovascular Surgery, Department of Surgery, Hospital de Clínicas, Universidade Federal do Paraná, Brazil.

**Keywords:** Cardiac Surgical Procedures, Longitudinal Studies, Medical Records, Risk Factors, Incidence, Length of Stay

## Abstract

**Objectives:**

The study aimed to determine the incidence of healthcare-associated infections (HAI) and their sites in a cardiac surgery service, as well as to determine if gender and age were risk factors for infection and to quantify mortality and increase in the hospital length of stay (LOS) due to HAI.

**Methods:**

Medical records of patients who underwent cardiac surgery from January 2012 to January 2018 were retrospectively analyzed. Data on age, gender, mortality, occurrence of HAI during hospitalization, and LOS were collected. Continuous variables were analyzed using Student's *t*-test, while categorical variables were compared using Fisher's exact test or chi-square test.

**Results:**

Among the 195 patients available, the HAI rate in our service was 22.6%, with female gender being a risk factor for infections (odds ratio [OR]=2.23; *P*=0.015). Age was also a significant risk factor for infections, with a difference in the mean age between the group with and without infection (*P*=0.02). The occurrence of an infectious process increased the LOS in 14 days (*P*<0.001) and resulted in higher mortality rates (*P*=0.112). A patient who has HAI was approximately 19 times more likely to remain hospitalized for more than nine days (*P*<0.001).

**Conclusion:**

Age and gender were risk factors for the development of HAI and the occurrence of an infectious process during hospitalization significantly increases the LOS. These findings may guide future actions aimed at reducing the impact of HAI on the health system.

**Table:** 

Abbreviations, acronyms & symbols	
COPD	= Chronic obstructive pulmonary disease
HAI	= Healthcare-associated infections
LOS	= Length of stay
MRV	= Myocardial revascularization
OR	= Odds ratio
SSI	= Surgical site infection

## INTRODUCTION

Healthcare associated infections (HAI) are one of the biggest challenges during healthcare for those who undergo a surgical procedure. There has been an elevation in life expectancy in recent decades and the rise in the incidence of noncommunicable diseases such as hypertension, chronic kidney disease and chronic obstructive pulmonary disease (COPD). These two processes affect not only the public administration, but also the surgical field, because they elevate the risks and costs of the procedure, as well as the increased length of stay (LOS) and the probability of infections^[1,2]^.

The main classes of complications resulting from cardiovascular surgery are cardiac, pulmonary, renal, neurological, infectious, hematological, digestive and hydroelectrolytic^[3]^. Although cardiovascular surgery represents a type of operation with low infectious rate, infectious processes are usually among the main complications described^[4-6]^. The rates of HAI in cardiac surgery vary between 6 and 24%^[3,6,7]^. Pneumonia, surgical site, and urinary tract are the most common sites of infection^[3]^. The age and gender of patients, the presence of comorbidities and the type of procedure performed seem to be risk factors for the development of infections^[7-9]^. The LOS of individuals who develop HAI is significantly increased compared to those without infection^[6,7]^. This excess of time that the patient remains in the hospital environment generates negative impacts for both the user and the health system as a whole, with gradually higher costs^[10]^.

This study aimed to determine the incidence of HAI and their sites in a cardiac surgery service, as well as to determine if gender and age were risk factors for infection and to quantify mortality and increase in the LOS due to HAI.

## METHODS

An observational cross-sectional study performed at the Cardiovascular Surgery Service of the Hospital de Clínicas of Universidade Federal do Paraná (HC-UFPR), Brazil. The study was completed with full approval of the Committee for Ethics in Research with Human Beings from the Hospital de Clínicas (HC-UFPR), CAAE: 81928918.6.0000.0096.

The population consisted of patients who underwent cardiac surgery from January 2012 to January 2018, who met the following criteria: 18 years or older; cardiac surgery by longitudinal median sternotomy; undergo at least myocardial revascularization (MRV), valve replacement or repair, closure of interatrial communication or partial pericardiectomy. Exclusion criteria were: insufficient or discordant data in medical records, patients transferred to other healthcare services, patients admitted with infectious disease or concomitant surgeries of other specialties during the same hospitalization.

Data collected from medical records included: age, sex, type of procedure performed, occurrence of HAI, mortality and site(s) of infection (if relevant), and LOS, considered from the first postoperative day. LOS was accounted following this pattern to standardize data, since delays could occur in the acquisition of surgical material or to schedule the surgery, which would be a bias for the LOS in some patients. The infection sites were subdivided into pneumonia, urinary tract infection, surgical site infection (SSI), and others (bacteremia, catheter-related, and colitis).

Data were collected and stored in a Microsoft Excel spreadsheet. Data analysis was performed using IBM SPSS v.22.0 software. Results were expressed as mean, median, minimum and maximum values, and standard deviation (quantitative variables) or by frequencies and percentages (qualitative variables). Student's t-test revealed whether the mean age or mean LOS between the group with and without HAI were statistically significant. Fisher's exact test showed the relationship between female gender and the occurrence of HAI, as well as the relationship of nosocomial infection with LOS >9 days and mortality. Multiple regression analysis compared the combined influence of age and sex on the incidence of HAI. Chi-square test was used to correlate age ranges and the incidence of infections. Results were considered significant at a significance level of 5% (P<0.05), giving 95% confidence that the results were correct.

## RESULTS

A total of 214 patients were selected, of whom 195 were eligible for the study. Among the 195 patients analyzed, 114 (58.5%) were male, while 81 (41.5%) were female, with a mean age of 60.6±13.5 years (quartiles 53, 62, 71) and a mean LOS of 12.7±11.9 days. All patients underwent cardiac surgery, with 101 (51.8%) patients submitted to isolated MRV, 71 (36.4%) to isolated valve replacement/repair, 11 (5.6%) to MRV plus valve replacement, 7 (3.6%) to interatrial communication closure; 4 (2.1%) to MRV plus valve repair and 1 (0.5%) to partial pericardiectomy (Table 1).

**Table 1:** Epidemiological data of the patients.

Variables	n (%)
Gender	
Male	114 (58.5)
Female	81 (41.5)
Type of surgery	
Isolated MRV	101 (51.8)
Valve replacement/repair	71 (36.4)
Valve replacement + MRV	11 (5.6)
IAC correction	7 (3.6)
Valve repair + MRV	4 (2.1)
Partial pericardiectomy	1 (0.5)
Infection	
Yes	44 (22.6)
No	151 (77.4)

IAC=interatrial communication; MRV=myocardial revascularization

From surgical procedure to hospital discharge, 44 patients (22.6%) developed HAI. The main sites of infection were: surgical (45.5%), pulmonary (45.5%), urinary tract (11.4%), and others (11.4%). The mean age of those who developed infection was 64.5±12.1 years, compared with the average of 59.5±13.8 years of those without infection (Table 2). When evaluated in age groups, the incidence of infections was concentrated in the two categories: from 60 to 69 years and from 70 to 79 years, accounting for 68.2% of HAI cases (Table 3).

**Table 2:** Mean age and length of stay between groups with and without infection.

	Infection		*P*-value
Mean (SD)	Yes, n=44	No, n=151	
Age (SD)	64.5 (12.1)	59.5 (13.8)	0.02
LOS (SD)	23.7 (15.0)	9.5 (8.4)	< 0.001

LOS=length of stay; SD=standard deviation

**Table 3:** Preoperative clinical characteristics and length of stay >9 days between groups with and without infection.

	Infection		*P*-value	OR
Results	Yes, n=44 (%)	No, n=151 (%)		
Mortality	8 (18.2)	15 (9.9)	0.112	
Females	25 (56.8)	56 (37.1)	0.015	2.23
LOS ≥9 days	39 (88.6)	44 (29.1)	< 0.001	18.97
Age (years)			0.088	
<60	12 (27.3)	73 (48.3)		
60-69	15 (34.1)	40 (26.5)		
70-79	15 (34.1)	32 (21.2)		
>80	2 (4.5)	6 (4.0)		

LOS=length of stay; OR=odds ratio

The mean LOS was 12.7 days, with a mean LOS of 23.7 days in the group of patients with infection and a mean LOS of 9.5 days for those without infection (Table 2). Infection development was an independent risk factor to elevate the hospital stay by more than nine days, with an odds ratio of approximately 19 (Table 3).

Female gender and a LOS of more than nine days were positive risk factors for the development of infections (Table 3). Multiple regression was performed to verify whether the patient’s age and gender were able to predict the occurrence of HAI. The analysis revealed a statistically significant model (F[2, 192]=5.776; *P*=0,004; R^2^= 0.057). Age (β=-0.181; t=-2.570; *P*=0.011) and gender (β=0.0171; t=2.427; *P*=0.016) are predictors of infection. Mortality from surgical procedures was 11.8%, with 15 (9.9%) deaths in the group without infection and 8 (18.2%) deaths in the group with infection (Table 3). The main causes of death involved: cardiogenic shock in 8 patients (34.8%); sepsis in 6 patients (26%) .

## DISCUSSION

Cardiovascular surgery is generally considered a clean surgery, once the respiratory, digestive, and urinary tract are not involved during the surgical procedure and there is rarely a local infectious process^[1,11]^. Different studies have shown that HAI rates can range from 6% to 24%, depending on the characteristics of the services and patients, while the HAI rate in the cardiovascular surgery service of HC-UFPR is 22.6%^[3,6,7,12]^. Although within the expected percentage, HAI in our service is close to the upper limit of normality. High incidence of patients with elevated surgical risks, more complex and invasive procedures, failure to prevent nosocomial infections, and the profile of institutional microorganisms may have contributed to the high infection rates found.

Pneumonia and SSI corresponded to the main sites of infections, followed by urinary tract infection and others (bacteremia, catheter-related, and colitis)^[13]^. Over the last decade, pneumonia has been described as the main villain during the perioperative period (contrary to common sense, which selects SSI as the main complication in cardiovascular surgery), representing between 30 and 40% of postoperative HAI^[3,9,13-16]^.

Patient characteristics, such as COPD, advanced age and steroid use, and characteristics of the surgical procedure, such as length of surgery and cardiopulmonary bypass and number of red blood cell transfusions, contribute to the development of infections^[13,17]^. Median sternotomy itself produces postoperative pain, leading to changes in lung mechanisms, reducing the patient's ability to cough, breathe deeply or clear secretions, thus predisposing the development of pneumonia^[14]^. Another relevant risk factor is the time on mechanical ventilation. Considering that the maximum recommended time for intubation is 24 hours, a ventilation period between 24 and 48 hours is related to twice as much chance of developing pneumonia, whereas a time greater than 48 hours produces four times more chance of lung infection^[15]^.

There are several perioperative risk factors for SSI, including high plasma glucose levels, high body mass index, number of blood transfusions, and bilateral grafts^[18,19]^. Although the use of bilateral internal thoracic arteries is more beneficial than the unilateral approach, this procedure dramatically reduces sternal bone perfusion, increasing the likelihood of infection^[14,20,21]^. The present study demonstrates that isolated MRV is the most common procedure performed by our cardiovascular surgery service. Isolated MRV and isolated valve replacement/repair accounted for 88.2% of the procedures, confirming data from previous studies^[2,3,11]^.

The prevalence of patients over 60 years submitted to cardiac surgeries has increased over the last two decades^[10]^. Age was another predictive factor for the development of infections. There was a difference between the mean age of the groups, and the group without infection had a mean age of 59.5 years, while the group with infection had a mean age of 64.5 years. Studies have shown that older patients are more likely to develop infectious processes during surgical procedures^[7,12]^. Two factors can explain this phenomenon. First, metabolic alterations in the senescence process cause a reduction of functional reserves and progressive deterioration of vital functions, leading to difficulty of recovery after surgery^[22]^. Second, older people are more likely to undergo multiple and more complex procedures as a result of the vast array of comorbidities involved or a long period of illness to which they are exposed. Thus, the high incidence of infections in the elderly can be explained by the binomial which includes being more susceptible to more complex procedures and having greater difficulty in recovery after surgery.

Female gender proved to be a factor twice as strong for the development of infections. Women, especially those with larger breasts, are predisposed to greater inferolateral tension and consequent surgical wound dehiscence, with a larger collection of bacteria in folded regions, which may represent a characteristic that allows women to have more infections^[20,23]^. Other risk factors in women include a higher incidence of diabetes, hypertension, heart failure and vascular disease^[16,19]^. Comorbidities weaken the immune system and prompt inflammation in predisposed individuals, leading to a higher incidence of HAI. Combining age and gender, it was possible to establish that older women were more susceptible to infection.

The evaluation of the hospital mortality rate may represent an indicator of the quality of care provided to the patient. Postoperative mortality in cardiac surgery varies from 3% to 11% in different studies, influenced by age, metabolic condition of patients, and type of procedure performed^[12,17]^. Although our study could not establish a clear relationship between mortality rates and the occurrence of HAI, several studies have already done so^[13]^. The development of an infectious process causes a significant impact on a patient already very sensitized by surgery, making it harder for the immune system to fight the pathogen. In many situations, patient comorbidities, as diabetes mellitus and lung disease, influence the development of HAI and mortality^[17]^. There was no statistical significance related to HAI and death rates, and this can be explained by the fact that there were many cases of cardiogenic shock in both the control group and the group with infections. Low cardiac output syndrome requiring inotropic drugs has been described as the primary cause of in-hospital mortality by Soares et al.^[3]^

The occurrence of infections elevates the patients' LOS^[10,15]^. In our study, we demonstrated that patients who developed HAI remained 1.5 times longer than those without infection, a 14-day increase in hospitalization. Another way of analyzing the impact that the occurrence of infections has on LOS is to evaluate patients who remained more than nine days, considered as the ideal LOS for heart disease^[7,12]^. Thus, it was possible to notice that the occurrence of HAI increases by 19 times the probability of being hospitalized more than nine days. The extra time that the patient remains in the hospital environment not only brings negative impacts for its own health (with higher chance to develop other complications), but also for the health system as a whole, costing an average of 3.5 times more than hospitalization without HAI, reaching values close to 30 thousand dollars^[10,24]^.

This study aimed to evaluate the contribution of unmodifiable risk factors (age and gender) on the development of HAI. However, modifiable preoperative and operative characteristics may contribute to the incidence of infections. Alternatives to reducing the rate of infection are an accurate identification and correction of these factors. Routine aspiration of tracheal secretion, shorter mechanical ventilation time, adequate antibiotic therapy, and glycemic control may be feasible procedures to combat infections.

### Limitation

The present study has some limitations. The number of patients analyzed was significantly lower than studies conducted by other colleagues, although most of our results have followed the trend and significance of other publications^[7,12,25]^. Studies involving multiple centers may result in a larger patient population. In terms of preoperative predisposing factors, only age and gender were evaluated, but other patient characteristics may also influence the incidence of infections. Comorbidities, reduced ejection fraction, impaired renal function, and elevated body mass index have been described as predictive factors for HAI^[15,17]^. The same applies to perioperative factors such as surgery time, cardiopulmonary bypass time, and number of blood transfusions^[17]^. Therefore, further studies evaluating all these variables are needed to better categorize infections and their risk factors. Finally, an analysis and quantification of the real economic impact of increased LOS due to HAI would be crucial for the health system and its managers to gain insight into the costs required to treat these types of infections.

Studies analyzing the risk factors for HAI are essential for cardiovascular surgery services and healthcare institutions to know the predictive factors for the occurrence of infections among their patients and a measure of the impact that HAI has on LOS. Therefore, actions can be taken to minimize HAI rates in the future, bringing benefits to both patients and the health system.

## CONCLUSION

This study demonstrated that the infection rate in the cardiac surgery service is within the expectations of the world literature. SSI and pneumonia represented the most common sites of infectious disease involvement. Female gender and age over 60 years were positive risk factors for the development of HAI. Although it was not possible to correlate mortality rates with HAI, infectious processes play an important role in terms of postoperative deaths. The occurrence of infections increased the hospital LOS of patients by two weeks, representing higher expenses for the health system.

The results of this research are expected to guide further studies and serve as a basis for improving actions aimed at mitigating HAI rates, allowing the patient to remain as short as possible in the hospital environment and reducing healthcare costs.

**Table:** 

Authors' roles & responsibilities	
GBF	Substantial contributions to the conception or design of the work; or the acquisition, analysis, or interpretation of data for the work; drafting the work or revising it critically for important intellectual content; final approval of the version to be published
JCSD	Substantial contributions to the conception or design of the work; or the acquisition, analysis, or interpretation of data for the work; drafting the work or revising it critically for important intellectual content; final approval of the version to be published
LAM	Substantial contributions to the conception or design of the work; or the acquisition, analysis, or interpretation of data for the work; drafting the work or revising it critically for important intellectual content; final approval of the version to be published
